# Swedish prospective multicenter trial on the accuracy and clinical relevance of sentinel lymph node biopsy before neoadjuvant systemic therapy in breast cancer

**DOI:** 10.1007/s10549-017-4163-2

**Published:** 2017-02-17

**Authors:** Linda Zetterlund, Fuat Celebioglu, Rimma Axelsson, Jana de Boniface, Jan Frisell

**Affiliations:** 1 Department of Clinical Science and Education, Södersjukhuset, Karolinska Institutet, Stockholm, Sweden; 2 Department of Surgery, Södersjukhuset, Stockholm, 118 83 Sweden; 3 Department of Clinical Science, Intervention and Technology, Division of Radiography, Karolinska Institutet, Stockholm, Sweden; 4 Department of Radiology, Karolinska University Hospital, Huddinge, Stockholm, 141 86 Sweden; 5 Department of Molecular Medicine and Surgery, Karolinska Institutet, Stockholm, Sweden; 6 Department of Surgery, Capio St Göran’s Hospital, Stockholm, 112 81 Sweden; 7 Department of Breast and Endocrine Surgery, Karolinska University Hospital, Solna, 171 76 Sweden

**Keywords:** Sentinel lymph node biopsy, Breast cancer, Neoadjuvant systemic therapy, Pre-treatment, Identification rate, False negative rate

## Abstract

**Purpose:**

The timing of sentinel lymph node biopsy (SLNB) in the context of neoadjuvant systemic therapy (NAST) in breast cancer is still controversial. SLNB before NAST has been evaluated in few single-institution studies in which axillary lymph node dissection (ALND), however, was commonly not performed in case of a negative SLNB. We investigated the potential clinical relevance of SLNB before NAST by performing ALND in all patients after NAST.

**Methods:**

This national multicenter trial prospectively enrolled clinically node-negative breast cancer patients planned for NAST at 13 recruiting Swedish hospitals between October 2010 and December 2015. SLNB before NAST was followed by ALND after NAST in all individuals. Repeat SLNB after NAST was encouraged but not mandatory.

**Results:**

SLNB before NAST was performed in 224 patients. The identification rate was 100% (224/224). The proportion of patients with a negative SLNB before NAST but positive axillary lymph nodes after NAST was 7.4% (nine of 121 patients, 95% CI 4.0–13.5). Among those with a positive SLNB before NAST, 23.2% (86/112) had further positive lymph nodes after NAST.

**Conclusions:**

In clinically node-negative patients, SLNB before NAST is highly reliable. With this sequence, ALND and regional radiotherapy can be safely omitted in patients with a negative SLNB provided good clinical response to NAST. Additionally, SLNB-positive patients upfront will receive correct nodal staging unaffected by NAST and be consequently offered adjuvant locoregional treatment according to current guidelines pending the results of ongoing randomized trials.

## Introduction

Sentinel lymph node biopsy (SLNB) is gold standard for axillary nodal staging in early-stage breast cancer. Long-term follow-up has not shown any significant difference in survival or regional control when omitting axillary lymph node dissection (ALND) after a negative SLNB [1]. Also in larger tumors, SLNB has comparable accuracy [2].

Lately, indications for neoadjuvant systemic therapy (NAST) have been extended to not only encompass locally advanced but also early operable stages of the disease. Since then, only half of patients planned for NAST show nodal involvement at presentation, and an additional 20–40% will be downstaged to node negativity during treatment [3].

ALND is the traditional staging procedure in the neoadjuvant setting but is associated with significant arm morbidity which is further aggravated by regional radiotherapy [4]. To mitigate this, SLNB has been studied in several trials outlined below; the timing in relation to NAST, however, remains controversial.

SLNB before NAST has been evaluated in clinically node-negative (cN0) patients in a number of small single-center studies with excellent identification rates (IR). False negative rates (FNR) were as low as 0% in those few studies in which ALND was performed after a negative SLNB [5–7]. However, the majority of studies omitted ALND after a negative SLNB and reported the absence of axillary recurrences after a median follow-up period of 11–36 months [8–10], which is arguably short in the context of breast cancer.

SLNB after NAST has mostly been performed in clinically node-positive (cN1) patients at presentation [11] or included in meta-analyses reporting combined results for cN0 and cN1 patients [12, 13]. Classe et al. reported on a prospective multicenter study in which both IR and FNR for SLNB after NAST were better for patients with cN0 compared to cN1 disease at presentation [14].

SLNB before NAST in cN0 patients provides axillary staging unaffected by primary systemic therapy and can guide treatment decisions regarding appropriate chemo- and radiotherapy. According to the updated 2014 ASCO guidelines, as well as the NCCN guidelines from 2016, women with cN0 operable breast cancer may be offered SLNB either before or after NAST in the absence of evident axillary nodal disease [15, 16]. With the purpose of avoiding two surgical procedures and in order to take advantage of the nodal downstaging effect of NAST, SLNB after NAST has gained popularity. However, clinically node-negative patients with undiagnosed metastases upfront are at increased risk of a false-negative SLNB after NAST in at least 11% and consequently locoregional undertreatment [17]. Staging of the axilla upfront by ultrasound and fine needle aspiration, however, cannot replace SLNB as it is associated with a sensitivity of only 21–25% in finding axillary metastasis in clinically node-negative patients [18, 19].

Thus, the primary aim was to study the agreement of the SLNB result before NAST with the ALND result after NAST in cN0 breast cancer patients, irrespective of the result of the SLNB upfront. The secondary aim was to evaluate the feasibility and false negative rate of repeat SLNB.

## Methods

This Swedish prospective multicenter trial recruited consecutive patients with biopsy-proven invasive breast cancer planned for NAST from 20 invited hospitals, of which 13 actively recruited patients to the present arm of the trial between October 1, 2010 and December 31, 2015. Ultrasound of the axilla was performed and in case of suspicious lymph nodes, fine needle aspiration cytology (FNAC) was recommended. Patients were recruited into two arms depending on their axillary status pre-NAST.

Patients with proven axillary lymph node metastasis were directed into the second arm of this trial which will be reported separately.

In the here reported arm of the trial, only cN0 patients were eligible.

SLNB was performed before NAST and ALND after NAST in all patients. A repeat SLNB, regardless of the primary SLNB result, was encouraged in conjunction with ALND. Exclusion criteria were inflammatory breast cancer, allergic reactions to Patent Blue V or radiolabeled colloid, and inability to give informed consent.

For more details see Clinical.Trials.gov identifier NCT02031042.

### Lymphatic mapping technique

Preoperative lymphoscintigraphy was optional. Lymphatic mapping was performed with ^99m^Technetium-labeled nanocolloid, Patent Blue V, or a combination of both. The definition of a sentinel lymph node (SLN) was the hottest node, any node with more than 10% of the radioactivity of the hottest node, any blue node or clinically suspicious nodes on digital exploration.

### Surgery

Breast surgery was either breast-conserving surgery or mastectomy. All patients underwent a standard ALND of levels I and II after NAST.

### Neoadjuvant systemic therapy

Both neoadjuvant chemotherapy and endocrine therapy were eligible treatments. Standard chemotherapy regimens contained anthracyclines and taxanes and were given either according to regional guidelines or within current study protocols. Endocrine therapy consisted of aromatase inhibitors. Anti-HER2 therapy was given in combination with taxane-based chemotherapy. Altered or interrupted treatment was recorded together with the reason for disruption.

### Response evaluation

Clinical and radiological response was evaluated by comparing findings in the breast and axillary lymph nodes at diagnosis with those before definitive surgery. Classification was according to the UICC criteria [20] apart from radiological partial response which was defined as more than 30% decrease in tumor load measured on the greatest diameter according to the RECIST-criteria [21]. Pathologic response was graded as described by Sataloff et al. evaluating tumor (T) and nodes (N) separately [22], see Table 4.

Post-NAST stage classification (ypTNM) was based on the 7th edition of the AJCC staging system [23]. Pathologic complete response was defined as no residual invasive disease in the breast and axillary lymph nodes (ypT0/is ypN0). Presence of isolated tumor cells (ITC, ypN0(i+)) was not defined as nodal pCR [24].

### Pathologic assessment of lymph nodes

Lymph nodes were handled and assessed according to Swedish National Guidelines for Pathologists. All SLNs were fixed in formalin, sliced at 2 mm intervals, and embedded in paraffin. Each paraffin block was then sectioned at three 200 µm levels, and each level was stained with hematoxylin and eosin. If no cancer cells were detected, immunohistochemical staining with cytokeratin was recommended. SLN metastases were classified according to the 7th edition of the AJCC breast cancer staging manual [25].

### Definitions

Clinical tumor stage was based on pre-NAST radiological size measured by mammography or ultrasound. The identification rate was defined as the number of patients with a successfully identified SLN divided by the total number of patients in whom an SLNB was attempted. The term “false negative rate” (FNR) was here adapted to the neoadjuvant setting, and was defined as the proportion of patients with a negative SLNB pre-NAST but at least one positive axillary lymph node post-NAST, divided by all node-positive patients with an identified SLNB pre-NAST [26]. FNR in repeat SLNB was defined as the proportion of patients with a negative SLNB after NAST but at least one positive non-sentinel node after NAST, divided by all patients with at least one involved node among patients with at least one identified repeat SLN. Accuracy was defined as the proportion of patients with a true-positive or true-negative SLNB out of all patients with a successful SLNB.

### Statistical analysis

Sample size calculation was performed prior to the initiation of this trial. With an estimated 50% of all patients having a positive SLNB, and a proposed sample size of 200 patients, estimation of the FNR in SLNB before NAST is based on 100 individuals. If assuming a true “false negative rate” of SLNB before NAST of 8%, a power of 80% will then be achieved with reported confidence intervals (CI) of ±7 percentages.

Descriptive statistics are presented as median values with their ranges for continuous variables and as distributions with their percentages for categorical variables. Comparison of groups according to sentinel lymph node status was performed after exploring normal data distribution. For comparison of non-parametric continuous data, the Mann–Whitney *U* test was applied. For comparison of non-parametric categorical data, Fisher’s exact test was used. A *p* value of < 0.05 was considered statistically significant. The statistical software programme IBM SPSS Statistics for Windows Version 23.0 (Armonk, NY, USA) was used for all analyses.

## Results

### Patients

**Fig. 1:**
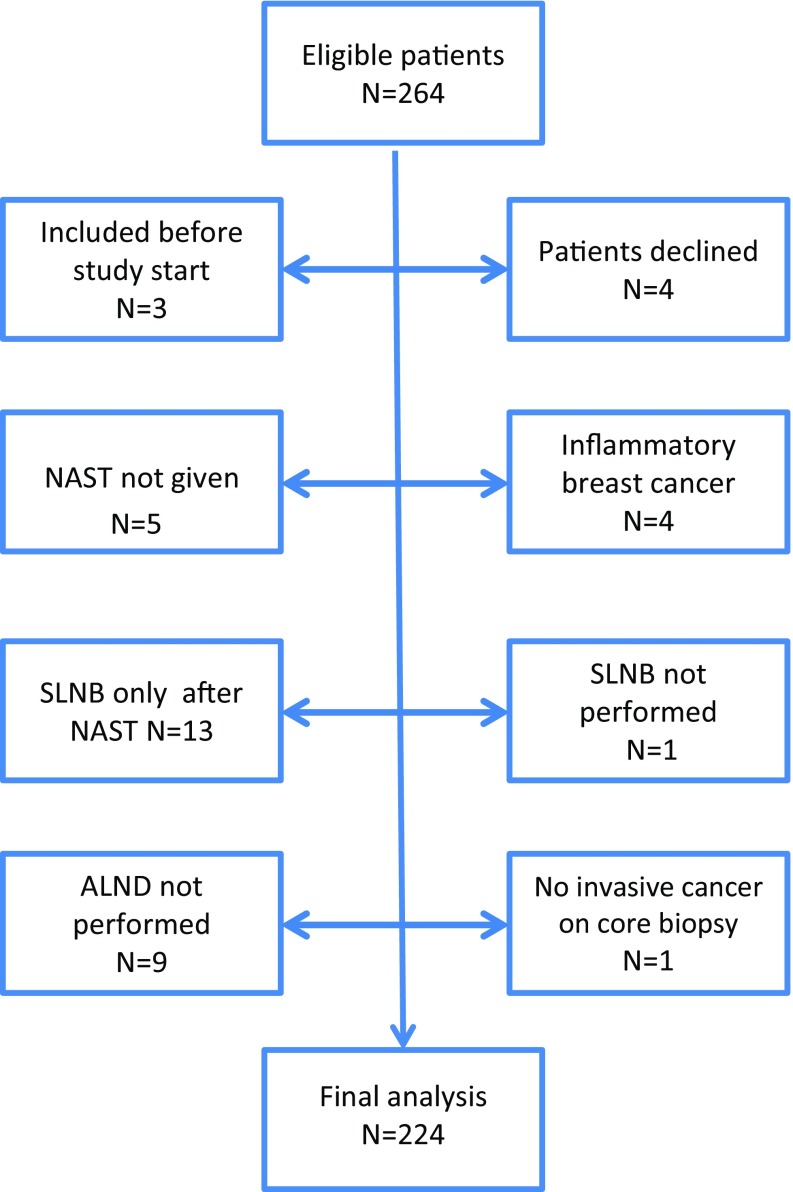
CONSORT diagram. NAST neoadjuvant systemic therapy, SLNB sentinel lymph node biopsy, ALND axillary lymph node dissection

Of an initial 264 eligible patients, 40 withdrew their consent or were excluded for other reasons. A CONSORT diagram is presented in Fig. 1. Thus, 224 patients from 13 recruiting hospitals operated by 67 surgeons were available for analysis. Median age was 47 years (range 22–78). Median radiological tumor size at diagnosis was 39 mm (range 9–127). An axillary ultrasound was performed in 97.3% (218/224) of the patients. Clinicopathologic and treatment characteristics of the trial population are reported in Table 1.

**Table 1:** Clinicopathologic and treatment characteristics of the trial population

	No. (%)
No. of patients	224
Median age, years	47, range 22–78
Radiological T stage	
T1	18 (8.0)
T2	149 (66.5)
T3	57 (25.4)
Histological type	
Ductal	181 (81.5)
Lobular	28 (12.6)
Other	13 (5.9)
Unknown	2 (0.9)
Nottingham histological grade	
I	5 (3.1)
II	76 (46.9)
III	81 (50.0)
Unknown	62 (27.7)
ER positive	137 (61.2)
PR positive	102 (45.5)
HER2-positive	72 (32.3)
Unknown	1 (0.4)
Neoadjuvant therapy	
Anthracycline plus taxane	199 (88.8)
Anthracycline only	10 (4.5)
Other type	13 (5.8)
Aromatase inhibitor	2 (0.9)

*ER* estrogen receptor, *PR* progesterone receptor, *HER2* human epidermal growth factor receptor 2

### Treatment

The majority of patients received anthracycline plus taxane-based chemotherapy (199/224, 88.8%). Only two patients 0.9% (2/224) had neoadjuvant endocrine therapy. Neoadjuvant systemic therapy regimens are presented in Table 1.

In 22 patients, treatment was interrupted prematurely due to intolerable side effects (9), toxicity (6), tumor progress (5), lack of response (1), or partus (1). In 49 patients, treatment was altered due to intolerable side effects (27), toxicity (10), lack of response (8), or tumor progress (4). Of all HER2-positive patients, 94.4% (68/72) received targeted treatment, 16.2% (11/68) of whom received dual antibodies. Breast-conserving surgery was performed in 65 of 224 patients (29.0%).

### SLN detection before NAST

Lymphatic mapping was performed using dual mapping in 95.5% (213/223) of patients. At least one SLN was identified in all patients pre-NAST, with a median of two SLNs (range 1–11). Half of all patients had a positive SLNB (112/224), 85.7% of whom (96/112) had at least one macrometastasis (median 1, range 1–6). After NAST, the median number of retrieved axillary lymph nodes, including repeat SLNs if any, was 10 (range 1–31), and the median number of positive axillary lymph nodes was two (range 1–12). Almost 77% (86/112) of patients with a positive SLNB before NAST had no positive axillary lymph nodes after NAST.

### False negative rate

A comparison of SLN status before NAST and overall axillary lymph node status (including pre- and post-NAST) is presented in Table 2. Nine patients had a negative SLNB before NAST but at least one positive lymph node in the axilla after NAST, resulting in a FNR of 7.4% (95% CI 4.0–13.5). Among these cases, the maximum number of positive lymph nodes was two, and the median number of retrieved axillary lymph nodes after NAST was nine (range 5–12). Details on those nine patients are displayed in Table 3.

**Table 2:** Cross tabulation of SLN status before NAST and overall axillary nodal status

SLNB before NAST	Overall axillary nodal status^a^		
	Positive	Negative	Total
Positive	112	0	112
Negative	9	103	112
Total	121	103	224

^a^Lymph node status in SLNs before, SLNs after NAST if performed, and non-SLNs after NAST. Sensitivity 92.6% (112/121), specificity 100.0% (103/103), and accuracy 96.0 % (215/224). SLN sentinel lymph node, SLNB sentinel lymph node biopsy, NAST neoadjuvant systemic therapy

**Table 3:** False-negative sentinel lymph nodes before NAST and corresponding axillary nodes after NAST

Patient number	Number of SLNs before NAST	Repeat SLNB attempted and nodes retrieved	Metastases in repeat SLNB	Metastases in non-sentinel nodes	Total number of axillary nodes after NAST	Total number of axillary metastases after NAST
13	2			N1	10	1
20	2	Yes, one	N0	N1	7	1
87	2			N1	12	1
95	1	Yes, one	N1	N1mi	9	2
108	1			N1	9	2
167	3	Yes, zero	Not identified	N1mi	5	1
196	1	Yes, two	N1	N0	9	1
408	4			N1	10	2
439	1			N1	8	2

*SLN* sentinel lymph node, *SLNB* sentinel lymph node biopsy, *NAST* neoadjuvant systemic therapy, *N1* macrometastasis, *N1mi* micrometastasis

There was no significant difference between patients with a false-negative compared to a true-positive or true-negative SLNB regarding any of those factors listed in Table 1. Only 22.2% of patients with a false-negative SLNB upfront achieved a complete clinical response (best clinical or radiological) in the breast, compared with 42.3% in the true-positive/true-negative group (*p* = 0.089). For complete pathologic response in the breast, the corresponding figures were 11.1 and 30.7%, respectively (*p* = 0.036), see Table 4.

**Table 4:** Comparison of tumor response between false-negative and true-positive/true-negative SLNs upfront

	True pos and true neg (%)	False neg (%)	*p*
No. of patients	215	9	
Best clinical or radiological response (ycT)			
Complete response	91 (42.3)	2 (22.2)	
Partial response	101 (47.0)	6 (66.7)	
No change	21 (9.8)	0 (0)	
Progress	2 (0.9)	1 (11.1)	0.089
Pathological response (ypT)			
Sataloff T–A	66 (30.7)	1 (11.1)	
Sataloff T–B	77 (35.8)	1 (11.1)	
Sataloff T–C	56 (26.0)	5 (55.6)	
Sataloff T–D	16 (7.4)	2 (22.2)	0.036

Complete response: The disappearance of all known disease, Partial clinical response: 50% or more decrease in total tumor load, No change: A 50% decrease in total tumor size cannot be established nor an increase of 25%, Progressive disease: 25% or more increase in size of one or more measurable lesions. Partial radiological response according to RECIST-criteria: 30% or more decrease in the sum of the longest diameter (LD) in target lesions, taking as reference the baseline sum LD. Sataloff T–A: Total or near total therapeutic effect, Sataloff T–B: >50% therapeutic effect but less than total or near total, Sataloff T–C: <50% therapeutic effect, but effect evident, Sataloff T–D: NO therapeutic effect. SLN sentinel lymph node

Of all patients with a negative SLNB before NAST, 92.0% (103/112) remained node-negative after NAST including one patient with ypN0(i+). A complete pathologic response in the breast (ypT0/is) was achieved in 33.9% (38/112), and a complete pathologic response in both axillary lymph nodes and breast (ypCR) was achieved in 33.0% (37/112). Among patients with a positive SLNB before NAST, 76.8% (86/112) had only negative nodes after NAST including two patients with ypN0(i+). A complete pathologic response in the breast (ypT0/is), and in both breast and axillary lymph nodes (ypCR), was achieved in 25.0% (28/112; *p* = 0.19) and 24.1% (27/112; *p* = 0.18), respectively.

### Repeat SLNB after NAST

In 98 patients, a repeat SLNB was attempted after NAST. Dual mapping was performed in 86.7% (85/98). In 69.4% (68/98), at least one SLN was identified. The median number of SLNs retrieved was 1 (range 1–5). The FNR for repeat SLNB was 25.0% (3/12). A comparison of SLN status after NAST and corresponding non-SLNs after NAST is presented in Table 5.

**Table 5:** Cross tabulation of repeat SLNB and axillary-involved nodes after NAST

Repeat SLNB	Overall axillary nodal status after NAST^a^		
	Positive	Negative	Total
Positive	9	0	9
Negative	3	56	59
Total	12	56	68

^a^Overall axillary lymph node status in SLNs and non-SLNs after NAST SLNB, sentinel lymph node biopsy, NAST neoadjuvant systemic therapy

## Discussion

We here present data from a prospective multicenter trial recruiting cN0 breast cancer patients planned for NAST at 13 Swedish hospitals. The excellent IR agrees with earlier studies evaluating SLNB before NAST [5–10] and confirms that the SLNB concept works well both in high- and low-volume hospitals. The high IR is probably due to a high rate of dual tracer use, underlining this method as the recommended technique. The much lower IR in repeat SLNB after NAST may reflect obstruction of lymph vessels with inflammatory debris secondary to NAST and postoperative scarring; this corresponds well with the results of the German four-armed SENTINA study in which the repeat SLNB IR was only 60.8% [26]. There is a possibility that the true IR might be even lower than reported in our trial since it cannot be ruled out that the repeat SLNB may have been identified only on the excised ALND specimen ex vivo instead of prior to ALND.

The proportion of patients with a negative SLNB before NAST but positive axillary lymph nodes after NAST was 7.4% in this trial, in which ALND was performed in all patients irrespective of the result of the SLNB upfront. This is comparable to the FNR in early-stage breast cancer [27], even though it cannot be ruled out that nodal metastases could have developed during the course of NAST in our trial, and thus, a direct comparison may be difficult. It should also be taken into account that the confidence interval around the point estimate is rather broad with 224 evaluated patients, as pointed out in the sample size calculation. In earlier publications, validating SLNB before NAST by post-NAST ALND, the FNR was 0%; however, these were all small single-institution studies at dedicated centers [5–7]. Based on the larger sample size and multicenter design in our trial we would suspect the true FNR to be closer to 7% than to 0% despite some uncertainty in the estimation. A false-negative SLNB, if not followed by an ALND, leads to incorrect nodal staging and inappropriate decision-making regarding adjuvant locoregional therapy. These individuals run the risk of being undertreated since an ALND will not be performed and adjuvant regional radiotherapy is unlikely to be recommended.

In our trial, one of the nine patients with a false-negative SLNB progressed clinically and/or radiologically during NAST, which was consequently interrupted after three cycles. Fewer patients with a false-negative SLNB tended to achieve a complete clinical response in the breast, and significantly fewer had a complete pathological response in the breast than those with a true-positive or true-negative SLNB. We therefore conclude that the decision to omit ALND after a negative SLNB upfront should be reconsidered if the clinical and/or radiological response has been poor. The axillary tumor burden, however, was low with a maximum of two macrometastases in the completion ALND after NAST, and it is thus unclear whether false negativity translates into a higher incidence of locoregional recurrences (LRR).

The median number of retrieved axillary lymph nodes after NAST was ten in this trial which is rather low but corresponds with earlier reports [28, 29]. We have no reason to interpret these numbers as inadequate axillary dissections. We rather believe they represent treatment effects and possibly technical challenges in analyzing the axillary pathology specimens after NAST.

The proportion of patients in our trial with a false-negative repeat SLNB was 25%, which is much lower than the 51.6% seen in arm B in the prospective four-armed SENTINA study by Kuhn et al. [26]. Contradictory to these results, Khan et al. reported on a FNR of only 4.5%, but repeat SLNB was only performed in 18 out of 33 individuals [30]. We therefore agree, despite relatively few patients in our trial, with the conclusion of Kuhn et al. in that a repeat SLNB cannot be recommended.

There is a lack of prospective data on LRR after NAST, especially after SLNB as the only staging procedure. In a recent retrospective study, clinically node-negative patients after NAST (ycN0) with a negative SLNB after NAST and no ALND were evaluated after five years of follow-up. Patients being cN0 or cN1/N2 before NAST had equally good overall survival. Only one patient developed a regional recurrence in the cN1/N2 group. In cN1/N2 patients with residual tumor burden in the breast, however, a negative SLNB after NAST had no influence on survival. The authors discussed if this was a consequence of higher false negative rates in this group with residual disease in the breast [31].

In early-stage breast cancer, the locoregional recurrence rate after a negative SLNB without ALND is low [32] despite false negative rates of 5–10% [27]. Also with a limited tumor burden, patients randomized to no ALND after a positive SLNB do not have a worse outcome than patients with an ALND performed [33, 34]. Even though these studies were underpowered to detect small yet clinically relevant differences, and only patients with breast-conserving surgery who received whole-breast adjuvant radiotherapy were eligible, they have resulted in significant practice changes. Translated into the neoadjuvant setting, these results would appear to support the use of SLNB before NAST and the omission of ALND in the case of SLN metastases in breast-conserving surgery, as 96–97% of patients in above-mentioned trials received adjuvant chemotherapy. They are not, however, adapted to support the omission of an ALND after a positive SLNB after NAST. Results from trials regarding this specific situation are still pending. On the other hand, patients fulfilling Z0011 criteria before NAST may, if SLNB is delayed until after NAST, remain undetected; some of them will convert into SLN-negative cases with an increased inherent FNR [17], others will remain SLN positive and, according to most current guidelines, undergo ALND. The first scenario results in a clear risk of the omission of locoregional treatment, and the second in unnecessarily extensive axillary surgery.

In cN0 patients planned for NAST, the timing of SLNB can be either before or after NAST. According to the ASCO guidelines from 2014 and the NCCN guidelines from 2016, both alternatives are valid [15, 16]. The advantages in performing SLNB upfront is that IR is excellent and nodal staging unaffected by NAST [26]. A correct nodal staging before NAST may help in deciding on optimal chemotherapy before and the most adequate locoregional treatment after NAST. However, two surgical interventions are mandated. SLNB after NAST has the advantage of only one operation, and more patients can be spared an ALND due to nodal downstaging in 20–40% [3]—if ALND is omitted in SLN-negative cases after NAST. The disadvantages, however, are lower identification rates and higher false negative rates after NAST and uncertainty on pre-treatment nodal stage, making decisions on axillary surgery and adjuvant radiotherapy more difficult with an obvious risk of undertreatment [17]. There are two ongoing randomized trials that will hopefully offer some answers to these questions [35]. Until then, performing SLNB upfront in clinically node-negative patients seems a safe and clinically relevant alternative.

## Conclusion

In clinically node-negative patients, a completion ALND can be safely omitted if SLNB before NAST is negative provided good clinical tumor response to NAST. Those patients with SLNB metastases upfront will receive nodal staging unaffected by NAST and be consequently offered adjuvant locoregional treatment according to current guidelines without the risk of undertreatment. They may also be enrolled into the Swedish-based SENOMAC trial, randomizing clinically node-negative patients with up to two positive SLNs to completion ALND or no further axillary surgery. A repeat SLNB is not recommended due to low identification rates and high false negative rates.
